# Effects of diesel exhaust particle exposure on a murine model of asthma due to soybean

**DOI:** 10.1371/journal.pone.0179569

**Published:** 2017-06-19

**Authors:** Daniel Alvarez-Simón, Xavier Muñoz, Susana Gómez-Ollés, Miquel de Homdedeu, María-Dolores Untoria, María-Jesús Cruz

**Affiliations:** 1 Pulmonology Service, Hospital Universitari Vall d’Hebron, Barcelona, Spain; 2 Medicine Department Universitat Autònoma de Barcelona, Barcelona, Spain; 3 CIBER Enfermedades Respiratorias (Ciberes), Barcelona, Spain; 4 Department of Cell Biology, Physiology and Immunology, Universitat Autònoma de Barcelona, Barcelona, Spain; Centre National de la Recherche Scientifique, FRANCE

## Abstract

**Background:**

Exposure to soybean allergens has been linked to asthma outbreaks. Exposure to diesel exhaust particles (DEP) has been associated with an increase in the risk of asthma and asthma exacerbation; however, in both cases the underlying mechanisms remain poorly understood, as does the possible interaction between the two entities.

**Objective:**

To investigate how the combination of soybean allergens and DEP can affect the induction or exacerbation of asthma in a murine model.

**Methods:**

BALB/c mice received intranasal instillations of saline, 3 or 5 mg protein/ml soybean hull extract (SHE), or a combination of one of these three solutions with DEP. Airway hyperresponsiveness (AHR), pulmonary inflammation in bronchoalveolar lavage, total serum immunoglobulin E and histological studies were assessed.

**Results:**

A 5 mg protein/ml SHE solution was able by itself to enhance AHR (p = 0.0033), increase eosinophilic inflammation (p = 0.0003), increase levels of IL-4, IL-5, IL-13, IL-17A, IL-17F and CCL20, and reduce levels of IFN-γ. The combination of 5 mg protein/ml SHE with DEP also produced an increase in AHR and eosinophilic inflammation, but presented a slightly different cytokine profile with higher levels of Th17-related cytokines. However, while the 3 mg protein/ml SHE solution did not induce asthma, co-exposure with DEP resulted in a markedly enhanced AHR (p = 0.002) and eosinophilic inflammation (p = 0.004), with increased levels of IL-5, IL-17F and CCL20 and decreased levels of IFN-γ.

**Conclusions & clinical relevance:**

The combination of soybean allergens and DEP is capable of triggering an asthmatic response through a Th17-related mechanism when the soybean allergen concentration is too low to promote a response by itself. DEP monitoring may be a useful addition to allergen monitoring in order to prevent new asthma outbreaks.

## Introduction

Several asthma epidemics due to soybean dust inhalation have been described in Spain and elsewhere [1–3]. The first reported epidemics occurred in Barcelona where soybean dust released during the unloading of these legumes from ships to silos caused asthma epidemics among residents of the neighborhoods closest to the harbor [1,2,4]. In this city, the control measures adopted to avoid these outbreaks included the reduction of allergen emission levels by the installation of filter bags, the establishment of threshold values compatible with health, and the daily assessment of the emission and dispersion of the allergen to keep levels below these thresholds [5]. However, in the determination of the threshold values the possible effect of pollution in combination with soybean allergens was not taken into account.

It is known that airborne particulate matter (PM), a major component of air pollution, may have direct effects on the pulmonary system, including the induction of inflammatory responses. Airborne PM has been related to an acute increase in the incidence of asthma in urban areas, particularly the fine and ultrafine particles emitted by vehicular traffic [6,7]. Diesel exhaust particles (DEP), the main contributor to traffic PM [6], have a potential enhancing effect on responses to inhaled allergen exposure, and may also induce sensitization to neoallergens in human and animal models [8]. Several mechanisms through which DEP could enhance sensitization to aeroallergens have been proposed [6,8,9]. Animal model studies suggest that exposure to DEP provokes allergic inflammation with Th2 and Th17 phenotypic differentiation, and that, in this differentiation, a specific role is played by environmentally persistent free radicals and polycyclic aromatic hydrocarbon fractions [7,8,10]. Attempts to explain the participation of DEP in the pathogenesis of asthma have suggested a role for oxidative stress and immune dysregulation, but at present the mechanisms involved remain poorly understood.

The experimental modeling of allergic airway inflammation, particularly in murine models, has made a significant contribution to our understanding of asthma pathogenesis. Traditional protocols used ovalbumin combined with a potent adjuvant, but this approach results in an acute asthma-like phenotype that does not model the etiology and natural history of human asthma [11,12]. In order to achieve a better simulation of the chronic nature of human asthma, longer duration models have been developed that avoid the use of adjuvants and include more physiologically relevant antigens, like house dust mite (HDM) [11–13]. The repeated exposure of the airway to low levels of allergens via inhalation or intranasal instillation has been shown to perform better than acute models with regard to the reproduction of some of the hallmarks of human asthma, such as allergen-dependent sensitization, a Th2-dependent allergic inflammation characterized by eosinophilic influx, sustained airway hyperresponsiveness (AHR), and even airway remodeling [11,13–15].

The aim of the present study was to develop and standardize a novel murine asthma model with repeated exposure of the airway to low levels of soybean, a physiologically potent and relevant outdoor aeroallergen, and to assess the effect of DEP on this murine model with regard to the development of AHR, lung inflammation, and immunological response.

## Material and methods

### Animals

Female BALB/c mice (20 g, 6 weeks old) were obtained from ENVIGO (Udine, Italy). Mice were housed in filter top cages in a conventional animal house with 12 h dark/light cycles and received slightly acidified water and pelleted food (Teklad 2014, Harlan Laboratories, IN, USA) ad libitum. All experimental procedures were approved by the Ethical Committee for Animal Experiments of Hospital Universitari Vall d’Hebron.

### Soy hull extract

Soy hull extract (SHE) was obtained as previously described [16]. In brief, soybean hull proteins were extracted in 0.1 M NH_4_HCO_3_ buffer. The eluate was filtered, dialyzed in a 3.5 kDa cut-off membrane and lyophilized. The protein concentration in the extract was 33% as determined by the bicinchoninic acid (BCA) method (Pierce Chemical Co., Rockford, IL, USA) following the manufacturer’s instructions. All the experiments were carried out with the same SHE batch.

### Diesel exhaust particles

Diesel exhaust particles (Standard Reference Material (SRM) 2975) were purchased from National Institute of Standards Technology (NIST) (Gaithersburg, MD, USA).The reported mean diameter of these particles was 11.2±0.1μm by area distribution, and the surface area, as determined by nitrogen gas adsorption, was 0.538±0.006m^3^/cm^2^.

### Experimental design

**Fig 1:**
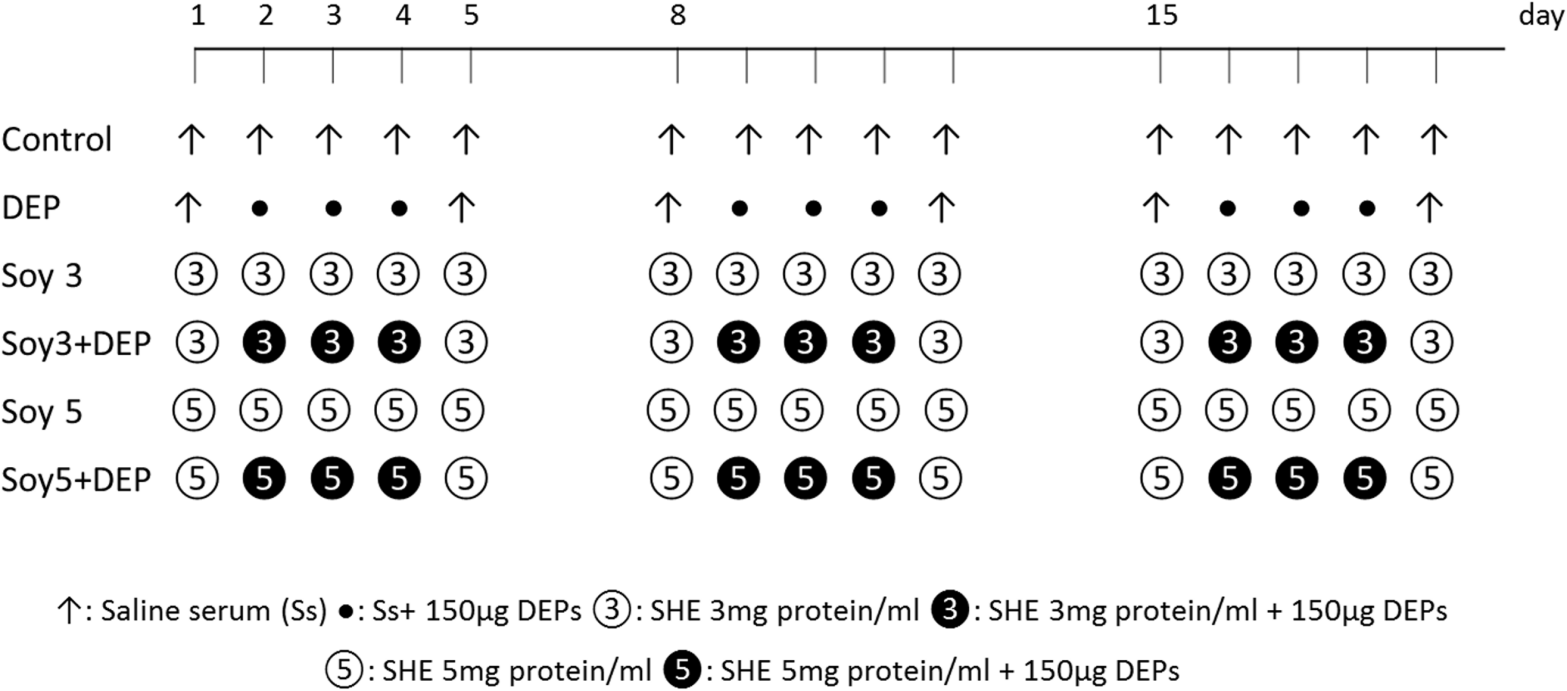
Schematic diagram of the experimental protocol and experimental groups. Six experimental groups were created. Female BALB/c mice received intranasal instillations of saline, 3 or 5 mg protein/ml soybean hull extract (SHE) on five consecutive days for three weeks. Solutions were administered three days a week, either alone or in combination with 150 μg of DEPs.

Six experimental groups were created. Soy hull extract was resuspended in sterile saline (0.9% NaCl, Fresenius Kabi, Barcelona, Spain) at two different concentrations of 3 and 5 mg protein/ml. Mice were exposed during five consecutive days over three weeks, under light anesthesia with isoflurane (Forane, Abbott Laboratories, Madrid, Spain), to either 20μl of saline, 20μl of SHE at 3 mg protein/ml concentration (Soy3), 20μl of SHE at 5 mg protein/ml concentration (Soy5), or a combination of one of these three solutions with 150 μg of DEP depending on the experimental group. In the groups receiving DEP, the supplement was administered three days a week for three weeks. A chart of the intranasal instillations of the six groups is shown in Fig 1. Each group comprised eight mice.

### Airway hyperresponsiveness

One hour after the last intranasal instillation, reactivity to methacholine was assessed invasively using a forced oscillation technique with the FlexiVent system (Flexivent, SCIREQ; Montreal, Canada). Mice were deeply anesthetized by an intraperitoneal injection of pentobarbital (70 mg/kg) (Nembutal, Abbot Laboratories, Spain). The trachea was exposed and, after tracheotomy, was connected to a computer-controlled ventilator. Airway resistance (R) was measured with a ‘‘snapshot” protocol and plotted against methacholine concentration (from 0 to 20 mg/ml), and the area under the curve (AUC) was calculated [17].

### Total serum immunoglobulin E (IgE)

After assessment of the methacholine test, blood was taken by cardiac puncture. Blood was centrifuged and serum samples were obtained and stored at -80°C for further analyses. Total serum IgE was measured using the Mouse ELISA IgE kit (Bethyl Laboratories, Inc., Montgomery, USA). Measurements were performed according to the manufacturer’s instructions.

### Bronchoalveolar lavage: Cell count

After blood sampling, bronchoalveolar lavage (BAL) was performed. The lungs were lavaged three times with 0.7 ml of sterile saline (0.9% NaCl). The first fraction recovered was stored separately and the following two fractions were pooled. The volume recovered was recorded. Total cells were counted using a hemocytometer and the BAL fluid was centrifuged (1000 g, 10 minutes, 4°C). The supernatant was frozen (-80°C) until further analyses. For differential cell counts, 100 μl of the resuspended cells (600000 cells/ml; 1400 g, 6 minutes) were spun (Cytospin 3, Shandon Thermo Scientific, Runcorn, Cheshire, UK) onto microscope slides, air-dried and stained with May-Grünwald for 5 min (QCA; Tarragona, Spain) and Giemsa for 15 min (Merck, Darmstadt, Germany). Cell counts were performed in 400 cells from each sample to determine the number of macrophages, eosinophils, neutrophils and lymphocytes.

### Bronchoalveolar lavage: Cytokines and chemokine

Levels of interferon-gamma (IFN-γ), interleukins-4 (IL-4), IL-5, IL-10, IL-13, IL-17A, IL-17F, IL-33, IL-31, IL-21, IL-22, IL-23 and the chemokine CCL20 were measured in the first fraction of undiluted BAL fluid by a mouse cytokine magnetic bead panel according to the manufacturer’s instructions (Bio-Plex X plex Custom Mouse Cytokine Assay, Bio-Rad Laboratories S.A.; Madrid, Spain).

### Lung pathology

After BAL, lungs were instilled with formaldehyde 3.7–4.0% until all lobes were deemed to be fully inflated by visual inspection. The tissues were formalin-fixed, paraffin embedded and cut in sections that were placed on slides. Evaluation of lung injury on slides stained by hematoxylin and eosin (H&E) was performed by an experienced pathologist in a blinded manner. A semi-quantitative scoring system was used to grade the severity and extent of inflammation on stained sections. Interstitial inflammatory infiltrate, peribronchial lymphoid activation and perivascular infiltrate were graded: 0 (normal) = absence of inflammatory cells; 1 (mild) = 1–2 layers of inflammatory cells; 2 (moderate) = 3–5 layers; 3 (severe) = more than 5 layers.

### Data analysis

All data are presented as medians with interquartile range, and were analyzed using the Kruskal-Wallis test with Dunn's multiple comparison posttest (Graphpad Prism 6.0, Graphpad Software Inc, San Diego, USA). A level of p≤0.05 (two-tailed) was considered significant.

## Results

### Airway hyperresponsiveness to methacholine

**Fig 2:**
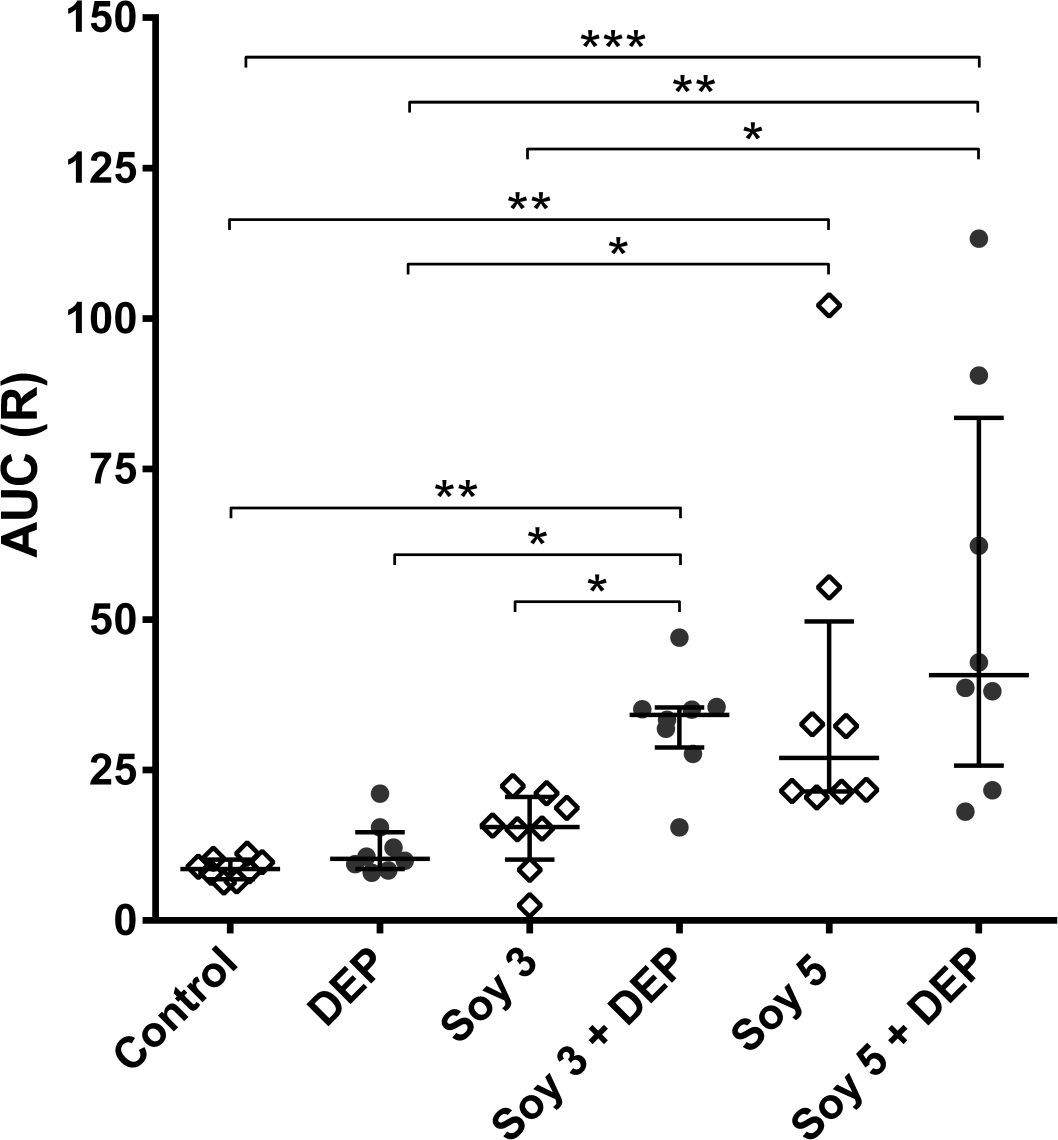
Airway hyperresponsiveness (AHR) to methacholine expressed as individual values of area under the curve (AUC) of resistance (R). Experimental groups are the same as in Fig 1. Median and interquartile range of individual values of AUC measured one hour after last intranasal instillation by the forced oscillation technique to increasing concentrations of methacholine. *p<0.05, **p<0.01; ***p<0.001.

To assess AHR to methacholine, AUC was calculated for each individual mouse in each experimental group (Fig 2). Exposure to DEP alone or to Soy3 did not induce any changes in AHR when compared with the control group; however, the combination of Soy3+DEP resulted in a markedly enhanced AHR compared to control, DEP and Soy3 groups (p = 0.002, p = 0.0116 and p = 0.048 respectively). Soy5 by itself significantly enhanced AHR compared with control and DEP (p = 0.0033 and p = 0.0277 respectively), while Soy5+DEP significantly increased AHR compared with control, DEP and also Soy3 (p = 0.0001, p = 0.0041 and p = 0.04 respectively). No significant differences were found between Soy3+DEP, Soy5 and Soy5+DEP.

### Total serum immunoglobulin E

**Fig 3:**
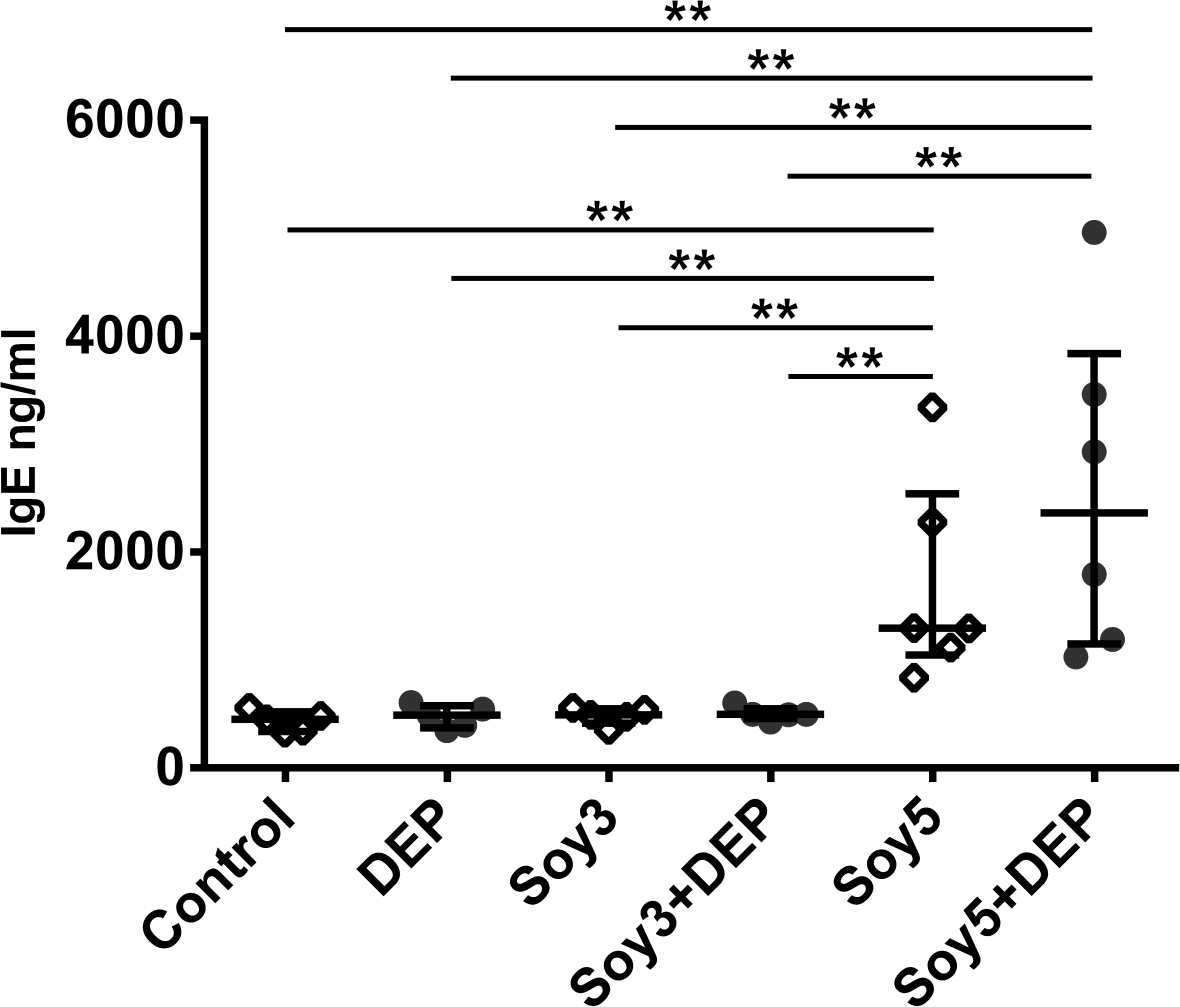
Total serum immunoglobulin (Ig)-E. Experimental groups are the same as in Fig 1. Median and interquartile range of total serum IgE. **p<0.01.

Total serum IgE in the six experimental groups are shown in Fig 3. Exposure to DEP, Soy3 or Soy3+DEP did not increase serum IgE levels, while Soy5 and Soy5+DEP groups showed significant increases in total serum IgE compared to control, DEP, Soy3 and also Soy3+DEP groups (Soy5: p = 0.0043, p = 0.0043 and p = 0.043 respectively; Soy5+DEP: p = 0.0043, p = 0.0043 and p = 0.043 respectively).

### Bronchoalveolar lavage: Cell count

**Fig 4:**
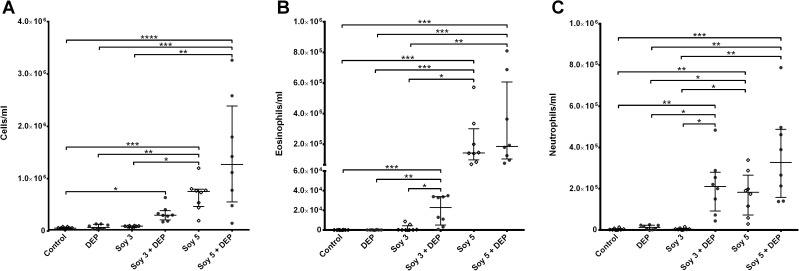
Number of total cells, eosinophils and neutrophil in one milliliter of BAL. Experimental groups are the same as in Fig 1. Median and interquartile range of total cell count (**A**), eosinophils (**B**) and neutrophils (**C**). *p<0.05; **p<0.01; ***p<0.001; ****p<0.0001.

Fig 4 shows the inflammatory cells in BAL. No differences were found in the total cell count when mice were exposed to DEP alone or Soy3 in comparison with control group. However, a significant increase in total cell count was found between Soy3+DEP and the control group (p = 0.0302). Soy5 alone and Soy5+DEP also showed increased total cell counts compared with control, DEP and Soy3 groups (Soy5: p = 0.0006, p = 0.0054 and p = 0.0252; Soy5+DEP p<0.0001; p = 0.0008 and p = 0.0044 respectively) (Fig 4A). Cell counts in the BAL showed differences in eosinophils and neutrophils between groups (Fig 4B and 4C). Similarly to total cell count, the DEP and Soy3 groups did not show any changes, but the Soy3+DEP showed increased numbers of eosinophils (p = 0.0004, p = 0.0015 and p = 0.04 respectively) and neutrophils (p = 0.002 and p = 0.0475 and p = 0.014 respectively) compared with control, DEP and Soy3 groups. Soy5 and Soy5+DEP groups also showed a significant increase in eosinophils (p = 0.0003, p = 0.0001 respectively), and neutrophils (p = 0.008, p = 0.0001) when compared both to controls, and to DEP (Eosinophils: p = 0.0007, p = 0.0003; Neutrophils p = 0.0486, p = 0.0066) and Soy3 (Eosinophils: p = 0.0106, p = 0.0047; Neutrophils p = 0.048, p = 0.0013).

### Bronchoalveolar lavage: Cytokines and chemokine

**Fig 5:**
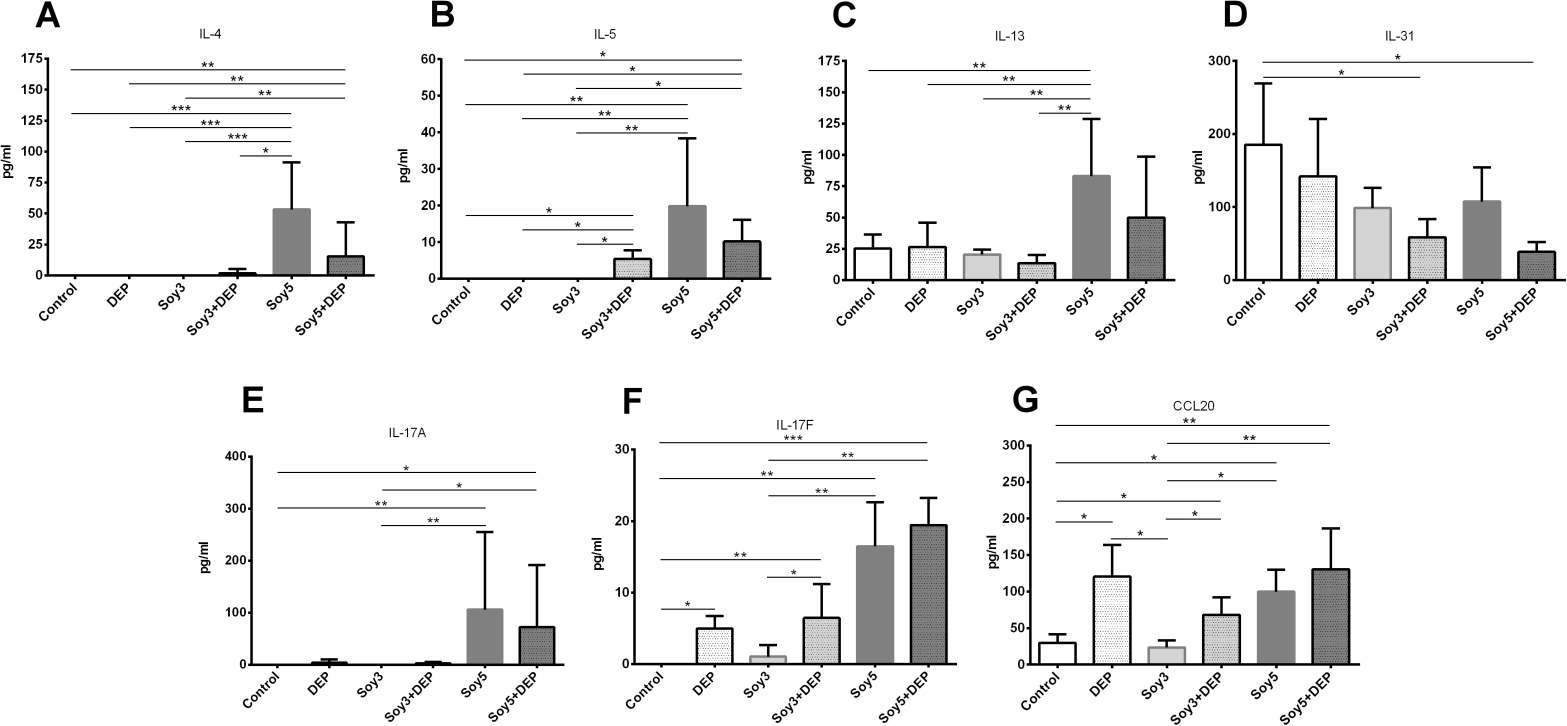
Levels of interleukin (IL)-4, IL-5, IL-13, IL-31, IL-10, IFN-γ, IL-17A, IL-17F and chemokine CCL20 in BAL fluid supernatant. Experimental groups are the same as in Fig 1. Median and interquartile range of(IL)-4 (**A**), IL-5 (**B**), IL-13 (**C**), IL-31 (**D**), IL-10 (**E**), IFN-γ (**F**), IL-17A (**E**), IL-17F (**F**) and chemokine CCL20 (**G**).

Measurement of the cytokines previously mentioned in BAL fluid revealed that the levels of IL-4 were increased in Soy5 and Soy5+DEP in comparison with control, DEP and Soy3 groups (Soy5: p = 0.001, p = 0.001, p = 0.001; Soy5+DEP: p = 0.0022, p = 0.0022, p = 0.0022 respectively), as well as compared with Soy3+DEP in the case of Soy5 (p = 0.0375) (Fig 5A). Levels of IL-5 (Fig 5B) showed an increase in Soy5 and Soy5+DEP, and also in Soy3+DEP (Soy5: p = 0.0082, p = 0.0082, p = 0.0082; Soy5+DEP: p = 0.0198, p = 0.0198, p = 0.0198; Soy3+DEP: p = 0.0495, p = 0.0495, p = 0.0495 respectively) when compared with control, DEP and Soy3. We only found significant changes in the levels of IL-13 (Fig 5C) and IL-10 (Fig 5E) in Soy5 group, where we found significant increases compared with controls, DEP, Soy3 and Soy3+DEP (p = 0.0022, p = 0.0087, p = 0.0022, p = 0.0022 respectively). The levels of IL-31 (Fig 5D) and IFN-γ (Fig 5F) showed decreases in both Soy3+DEP and Soy5+DEP groups and also, in the case of IFN-γ, in Soy3+DEP compared to controls (p = 0.0339, p = 0.013 respectively). IL-17A showed significant increases only in Soy5 and Soy5+DEP groups in comparison with controls and Soy3 (Soy5: p = 0.0029, p = 0.0029; Soy5+DEP: p = 0.015, p = 0.015 respectively) (Fig 5E). In contrast to IL-17A levels, both IL-17F (Fig 5F) and CCL20 (Fig 5G) levels were significantly increased in Soy5 and Soy5+DEP in comparison with controls but also in Soy3+DEP and DEP (Soy5: p = 0.0013, p = 0.0389, Soy5+DEP: p = 0.0003, p = 0.0084; Soy3+DEP: p = 0.0036, p = 0.0358; DEP: p = 0.0138, p = 0.0168 respectively). Furthermore Soy5 and Soy5+DEP and Soy3+DEP showed a significant increase in IL-17F (Soy5: p = 0.0066; Soy5+DEP: p = 0.0016; Soy3+DEP: p = 0.0311 respectively) in comparison with Soy3, while levels of CCL20 were significantly higher in Soy5 and Soy5+DEP, Soy3+DEP and also DEP (Soy5: p = 0.0246; Soy5+DEP: p = 0.005; Soy3+DEP: p = 0.02; DEP: p = 0.0103 respectively) compared to Soy3. Levels of IL-21, IL-22, IL-23, and IL-33 did not show statistical differences between groups. Levels of IL-10 and IFN-γ were undetectable or too close to the limit of detection to be analyzed.

### Lung histopathology

**Fig 6:**
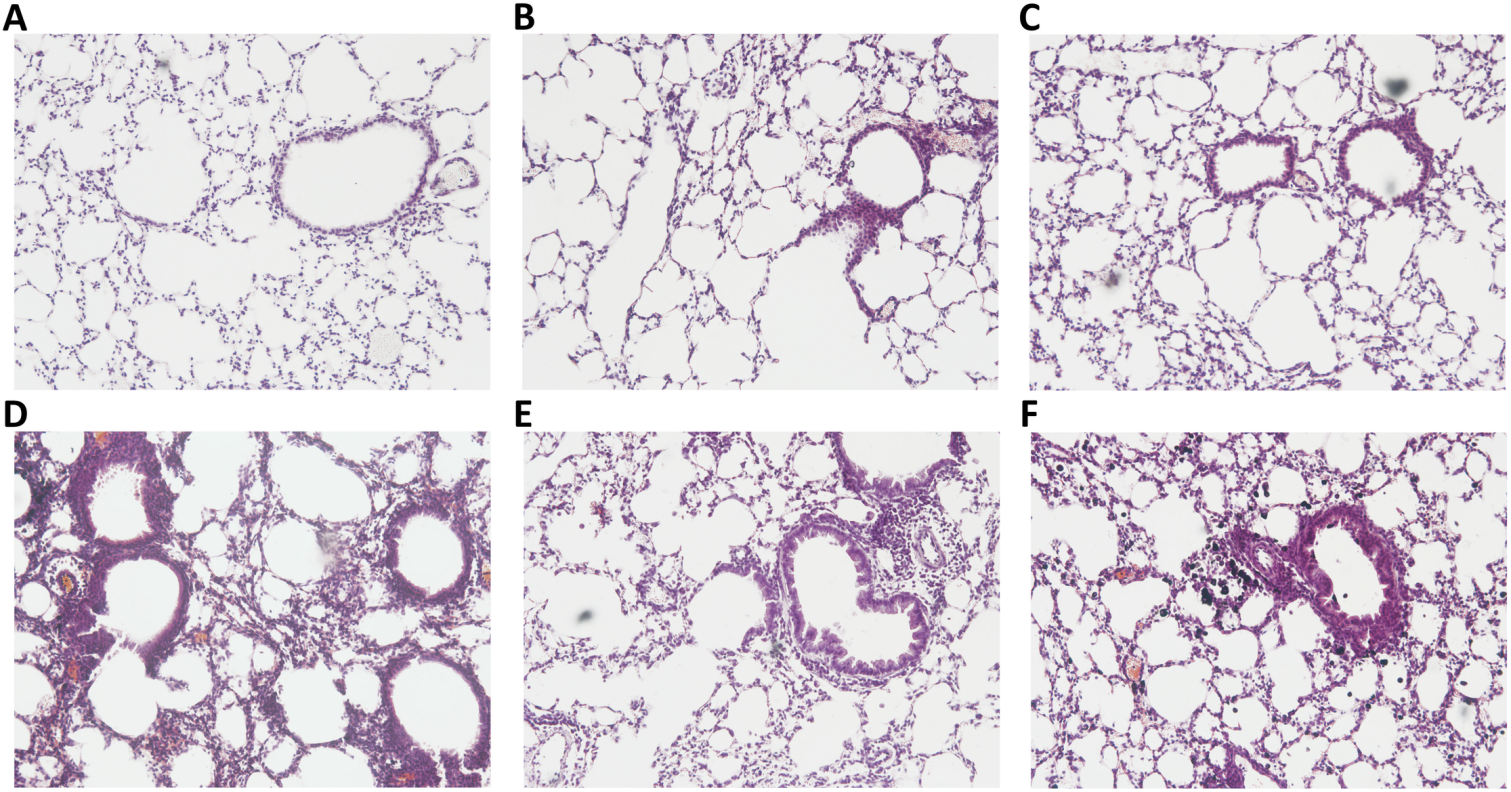
Lung histopathology. Representative images of hematoxylin and eosin stained histological lung sections. Slides photographed at 100x magnification. Experimental groups are the same as in Fig 1. (**A**) Control, (**B**) DEP, (**C**) Soy3, (**D**) Soy3+DEP, (**E**) Soy5, (**F**) Soy5+DEP.

The blinded histopathological examination of lung tissue sections revealed a mild to moderate (grade 1–2) interstitial, perivascular and peribronchiolar inflammatory infiltrate in the Soy5 (Fig 6E) and Soy5+DEP groups (Fig 6F). While no inflammatory infiltrate was observed in Soy3 group (Fig 6C), Soy3+DEP showed a mild to moderate (grade 1–2) interstitial inflammatory infiltrate, and a mild (grade 1) perivascular and peribronchiolar inflammatory infiltrate (Fig 6D). No inflammatory infiltrate was observed in the control group (Fig 6A), while the DEP group (Fig 6B) showed a moderate interstitial inflammatory infiltrate.

## Discussion

In this study, we assessed the effect of air pollution, in particular DEP exposure, on a new soybean asthma murine model. Our results show that the repeated intranasal administration of soybean, in the form of a sufficient concentration of SHE, triggers a severe asthmatic response with increased AHR, a robust inflammatory response in BAL, and increased levels of IgE, Th2-related cytokines such as IL-4 and IL-13, and also of Th17 cytokines. The results also demonstrate that when the concentration of SHE is too low to produce an asthmatic response by itself, DEP can synergize with SHE to produce a response. However, the immunological mechanism underlying the effect of the combination of lower SHE concentrations (3 mg/ml) with DEP seems to be Th17-related.

Asthma caused by soybean is a relatively well known entity, but in spite of its significance as a relevant outdoor allergen, little or no experimental work has been carried out to date to understand its airway immune-inflammatory responses. Nor, to our knowledge, has the interaction between soybean aeroallergens and DEP been adequately assessed: it has been studied mainly in ovalbumin (OVA) and house dust mite (HDM) murine models, but OVA is not a physiologically relevant allergen, and HDM is mainly an indoor allergen.

Our soybean asthma model showed that exposure to soybean aeroallergens in a sufficient concentration (5 mg/ml) leads to increased AHR, pulmonary inflammation with increases in both eosinophil and neutrophil counts and also higher levels of IgE. Nonetheless, our results also show that this response involves not just a Th2-driven inflammation with IL-4, IL-5, IL-13 and IL-10, but a more complex mechanism as well which, besides increases in Th2-related cytokines, also presents increased levels of Th17-related cytokines like IL-17A, IL-17F and the chemokine CCL20. Th17-mediated inflammation is increased in patients with severe asthma [18] and Th17 cells have been reported to be alternative drivers of severe asthma pathophysiology in addition to the Th2 pathway [18,19].

CCL20 is a chemokine derived from bronchial epithelial cells in response to several stimuli such as proinflammatory cytokines and ambient particulate matter, and is involved in the pathogenesis of asthma [20]. It is a functional ligand for CCR6, and via this receptor it is able to attract Th17 cells to the site of airway inflammation [20–22]. It has also been reported that the CCL20/CCR6 system may play a pivotal role in allergic airway responses such as AHR, airway eosinophilia, and production of IL-5 and IgE [20,21,23]. IL-17A and IL-17F are two proinflammatory cytokines, produced mainly by Th17 cells but also by other cells like bronchial epithelial cells [18,21]. They are known to recruit, activate and regulate the migration of neutrophils [21,22,24] and their increased levels in the lung of asthmatic patients are directly correlated with disease severity (i.e., increased AHR to methacholine) [24]. Animal model studies have also established a causative link between Th17 cells and increased levels of IL-17A and IL-17F with glucocorticoid-insensitive asthma [18,19,21,24].

The effect of the combined administration of an allergen with DEP on asthma and some of its hallmarks such as AHR and pulmonary inflammation has been described in previous studies using other murine models with different allergens and protocols. In acute models based on OVA administration [25] or chronic models with HDM [10,26] it has been observed that exposure to DEP cannot produce an asthmatic response and has no effect over AHR. Nonetheless, the effect of DEP on pulmonary inflammation and, more specifically, on neutrophilic inflammation is a controversial issue. DEP has been reported to cause an increase in pulmonary neutrophils [10,27,28]. However, exposure to DEP without any changes in neutrophil counts has also been described both in human exposure studies [29] and in animal studies [26,30,31].

The combination of DEP with an allergen may provoke a marked increase in AHR compared with exposure to the allergen alone [10,26,28,32]. Several studies have related allergen and DEP exposure with increases in pulmonary inflammation [6,28]. Increases in both neutrophil and eosinophil counts in BAL are a common feature in murine models exposed to DEP combined with allergens [6,26,28,33]. Muranaka *et al*. were the first to describe the effect of DEP and its combination with an allergen on the production of IgE [34]. Since then, the relationship between DEP exposure and IgE has been a controversial issue in the literature: while some authors observed significant increases in IgE levels [25,26,32,33], others reported no changes [6,28].

In our model, the combined administration of SHE with DEP produced different results depending on the allergen dose administered. The combination of Soy5, a SHE solution capable of producing an asthmatic response by itself, with DEP caused significant increases in AHR, pulmonary inflammation and IgE levels in comparison with the control group. In comparison with Soy5 alone, there were no significant changes in any of these asthma hallmarks, even though the asthmatic response seems to be stronger, with higher AHR, a greater degree of pulmonary inflammation, and a higher level of IgE. Nonetheless, the administration of Soy5+DEP results in a different cytokine profile: Soy5+DEP caused decreases in IFN-γ and IL-31 and increases in levels of IL-4, IL-5 and in all the Th17-related cytokines analyzed, but the levels of IL-13 and IL-10 presented no significant variations compared to controls. The decrease in both IFN-γ, a Th1 signature cytokine that can act as an inhibitor of Th17 differentiation [18,24], and IL-31, a Th2 cytokine whose production is induced by IL-4 [35], together with the absence of IL-13 and the increases in IL-17A, IL-17F and CCL20 compared to controls, resulted in a mixed Th2/Th17 response. These results, together with the increased levels of IL-17F and CCL20 promoted by exposure to DEP alone, support the activation of the Th17 response related to DEP exposure [10,36,37]. This partial downregulation of the Th2 response may be mediated by the counter regulation mechanism between the Th2 and Th17 pathways described by Choy et *al*. 2015 [19].

The Soy3+DEP combination produced significant increases in AHR and pulmonary inflammation not just compared to the control group, but also, and more importantly, compared to the Soy3 group. As stated above, the literature suggests that the immune mechanism underlying the effects of soybean aeroallergens on asthma onset and exacerbation and even on asthma outbreaks is an allergic asthma response based on allergen-driven Th2 inflammation, in which interleukins IL-4, IL-5, and IL-13 are the main mediators [38]. However, Soy3+DEP promotes AHR and both neutrophilic and eosinophilic pulmonary inflammation, without increasing the levels of IgE, and more importantly without the involvement of IL-4 and IL-13. The absence of the most important Th2 mediators, and the significant increases in Th17-related cytokines, suggest that a Th17-mediated response might be the mechanism underlying the increases in AHR and in both neutrophilic and even eosinophilic pulmonary inflammation. Th17 cells IL-17A and IL-17F are usually associated only with neutrophilic inflammation [18,21,24], but as our results and previous literature reports suggest, they may have multiple forms of biological activity over bronchial epithelial cells [18,20,21,39], airway smooth muscle cells, fibroblasts, endothelial cells [18,21] and even directly over eosinophils [18,21,40]. Eosinophilia of the airway is a phenomenon associated with Th2 allergen-driven inflammation and with the presence of IL-5 produced by Th2 cells, but in fact, the eosinophils themselves are among the main producers of IL-5 [40]. Besides, some authors propose that Th17 cells and their cytokine milieu are involved in the activation of eosinophil production, maturation, survival and cytolytic activity [19,40], probably through the recently described Th2-independent mechanism of eosinophil activation mediated by the production of GM-CSF by Th17 cells related to DEP exposure [41,42].

This study has some limitations. The first is in relation to the levels of total IgE in serum which were determined as a surrogate of specific SHE IgE levels. We cannot rule out the possibility that there might have been a production of low levels of specific IgE in the soy3 condition. Second, due to sample limitations, other relevant antibodies such as IgG1 or IgG2a were not measured. Finally, the absence of flow cytometry confirmation of the presence and frequency of Th2/Th17 and other cells like type 3 innate lymphoid cells limits our understanding of the underlying cellular mechanism and our ability to identify the cells responsible for the production of the cytokines measured in BAL. Moreover, information on other cytokines such as IL-6 or IL-1β which play an important role in the immunological pathways involved might provide a better understanding of the mechanisms related to asthma due to soybean and DEP.

To our knowledge, ours is the first study to standardize a murine model of asthma due to soybean and to assess the combined effect of soybean aeroallergens and DEP. Our experiments show that the continuous administration of soybean allergens at a certain concentration is capable of triggering an asthmatic response. In addition, we demonstrate that coexposure to soybean allergens and DEP results in a stronger asthmatic response, increasing airway hyperresponsiveness and pulmonary inflammation even when the concentration of soybean allergen is incapable of promoting an inflammatory response by itself. This mouse model provides evidence that the mechanism underlying soybean asthma is a mixed Th2/Th17 response, and also that DEP is capable of enhancing the allergenic effect of soybean through a Th17-mediated mechanism. These findings suggest that particulate matter monitoring as a surrogate of DEP exposure may be a useful addition to the allergen monitoring in the attempts to prevent new asthma outbreaks.

## Abbreviations

AHR: airway hyperresponsiveness
AUC: area under the curve
BAL: bronchoalveolar lavage
DEP: diesel exhaust particles
HDM: house dust mite
OVA: ovalbumin
PM: particulate matter
R: Airway resistance
SHE: Soy hull extract
